# Impact of Major Depressive Disorder on Prediabetes by Impairing Insulin Sensitivity

**DOI:** 10.4172/2155-6156.1000664

**Published:** 2016-04-28

**Authors:** Li Li, Richard Charles Shelton, Rachel Ann Chassan, John Charles Hammond, Barbara Ann Gower, Timothy W Garvey

**Affiliations:** 1 Department of Psychiatry and Behavioral Neurobiology, University of Alabama at Birmingham, 1720 University Blvd, Birmingham AL, 35294, USA; 2 Department of Nutrition Sciences, University of Alabama at Birmingham, Birmingham AL, 35294, USA; 3 Department of Nutrition Sciences, University of Alabama at Birmingham, and the Birmingham VA Medical Center, Birmingham AL, 35294, USA

**Keywords:** Insulin sensitivity, Prediabetes, Atherosclerosis, Phosphorylation, Cardiovascular diseases, Glucose tolerance test

## Abstract

Reports regarding the associations between major depressive disorder (MDD) and diabetes remain heterogeneous. Our aim was to investigate whether glucose homeostasis and insulin sensitivity were impaired in the MDD patients and its mechanisms. A total of 30 patients with MDD and 30 matched controls were recruited. The oral glucose tolerance test and dual-energy X-ray absorptiometry scan were performed in each participant. Insulin signaling in postmortem brain tissues from other depressive patients and controls (obtained from Alabama brain bank) was examined. Insulin sensitivity was reduced substantially in the MDD patients, however, the fasting and 2-h glucose concentrations remained within the normal range through compensatory insulin secretion. Despite increased insulin secretion, 1-h glucose concentrations in the MDD patients were significantly elevated compared with the controls. MDD patients had greater visceral fat mass but lower adiponectin levels compared with the controls. Furthermore, phosphorylated-AKT levels in insulin signaling were decreased in postmortem brain tissues in patients with MDD. These results suggest that MDD patients are at a greater risk for diabetes due to decreased insulin sensitivity, reduced disposition index, and impaired glucose tolerance as manifested by elevated 1-h glucose concentrations following an oral glucose challenge. Mechanistic studies reveal that decreased insulin sensitivity is associated with increased visceral fat mass, lower adiponectin levels and impaired insulin action in postmortem brain tissues in the MDD patients. Our findings emphasize the importance of screening depressive patients to identify susceptible individuals for developing future diabetes with the hope of improving their health outcomes.

## Introduction

Prediabetes is a state defined as impaired fasting glucose and/or impaired glucose tolerance [1]. The prediabetic state could last for years, and more than 30% of these individuals finally develop type 2 diabetes. Previous studies have shown that individuals with prediabetes not only have an increased risk of developing type 2 diabetes but also have a higher prevalence of cardiovascular diseases, including atherosclerosis, compared with normoglycaemic subjects [2]. Both cross-sectional and longitudinal studies have demonstrated that prediabetes is the result of decreased insulin sensitivity and/or the inability of the β-cells to adequately compensate through increased insulin secretion [3,4]. Therefore, effective strategies to improve insulin sensitivity and/or to increase insulin secretion could greatly decrease prediabetes and subsequent type 2 diabetes. Although mechanisms have not been fully elucidated, patients with major depression frequently develop impaired glucose tolerance [5,6].

Major depressive disorder (MDD) and diabetes are highly prevalent in the U.S. Approximately 16% of the U.S. adults will suffer from MDD at some point in their lives [7]. Over 6.5% of the U.S. adult population has been diagnosed with diabetes [8]. The etiology of type 2 diabetes involves a complex interaction between genetic, epigenetic and environmental factors [9-11]. It is well-established that the increasing prevalence of type 2 diabetes cannot be explained by genetics alone, and that environmental factors play a crucial role [9-11]. Psychosocial stress is believed to be one of the main environmental factors increasing the risk for type 2 diabetes [9-11]. Individuals with MDD tend to become less active and adopt a poor diet [9,10]. Thus poor health behaviors, including physical inactivity and caloric intake, and obesity in the MDD patients could induce insulin resistance, contributing to a higher risk for diabetes. Although cross-sectional studies have established the relationship between diabetes and depression, whether alterations in insulin sensitivity and insulin secretion are present in individuals with MDD is uncertain.

Insulin exerts its biological effects by binding to its specific tyrosine kinase receptors on the surface of target cells [12]. Activation of the receptor tyrosine kinase leads to its autophosphorylation and further phosphorylation of insulin receptor substrates (IRS), which serve as “docking” molecules, favoring the generation of intracellular signals. There are two main insulin intracellular signaling pathways: IRS-phosphatidylinositol 3-kinase (PI3K)-Akt pathway and the Ras-MAPK (MEK-ERK) pathway [12]. Insulin resistance refers to a decreased capacity of target cells to respond to normal levels of circulating insulin due to impairment of one or more signaling pathways. Studies found that defects in insulin signaling were selective with a dramatic reduction of insulin-stimulated IRS-PI3K-Akt pathway rather than the MEK/ERK pathway in type 2 diabetic patients [13].

The aim of present study was to investigate whether glucose metabolism and insulin sensitivity were impaired in the MDD patients, and to identify the potential mechanisms linking MDD with prediabetes and subsequent diabetes. The primary objective of this study was to evaluate the relationship between MDD, glucose homeostasis and insulin sensitivity using a 2-h oral glucose tolerance test (OGTT). Secondary exploratory analyses sought to better understand the possible mechanisms influencing insulin sensitivity in the depressive patients by measuring body composition using dual-energy X-ray absorptiometry (iDXA) and by investigating the insulin signaling pathway in postmortem human brain samples.

## Methods

### Subjects

All participants were recruited through the Outpatient Clinical Research Program of the Department of Psychiatry at the University of Alabama at Birmingham (UAB), in which study subjects were recruited from responses to local newspaper advertisements or from people attending clinics at the UAB. The project was approved by the UAB Institutional Review Board and in accordance with the Helsinki Declaration of 1975. All participants provided written informed consent prior to completing any research procedures. Participants included males and females between 19 and 55 years of age (Table 1). Participants were excluded if they: (1) had a known history of diabetes; (2) were taking medications known to affect glucose tolerance; (3) were pregnant or lactating; or (4) had a history of psychosis, bipolar disorder, or drug or alcohol abuse or dependence within 1 year prior to enrollment. Sixty seven participants were recruited, but 7 participants dropped out due to fear of needles, lack of transportation or loss of contacts. Thus 60 subjects completed the study and divided into two groups: subjects with- (MDD group, *n*=30) and without a diagnosis of MDD (control group, *n*=30). Diagnosis of MDD was confirmed using the Structured Clinical Interview for DSM-IV [14] or the Mini International Neuropsychiatric Interview [15]. The severity of MDD was determined using Beck depression scale. Family history of diabetes was collected by self-report.

### Glucose tolerance test

Glucose tolerance was classified by the American Diabetes Association criteria with a 75-g OGTT [16]. After 10-12h of overnight fasting, participants underwent a 2-h OGTT. Blood samples were drawn at 0, 0.5, 1 and 2-h for the determination of glucose and insulin levels. Glucose was measured by the glucose oxidase method using a glucose analyzer (Beckman Coulter Unicell DxC 800). Insulin was determined using a solid phase, enzyme labeled, chemiluminescence immunoassay (Siemens).

### Calculations

Fasting glucose and insulin concentrations were obtained from the baseline measurement of the OGTT. Incremental area under the curve (iAUC, concentration×time) for glucose response and insulin secretion during the 2-h OGTT was calculated using the trapezoidal method. In the present study, we used the homeostasis model assessment of insulin resistance (HOMA-IR) and quantitative insulin sensitivity check index (QUICKI) as estimates of insulin sensitivity since both have proved to be a robust tool for the surrogate assessment of insulin sensitivity [17,18]. Using the OGTT data, both HOMA-IR and QUICKI were calculated for each participant as previously described [17,18]. The insulinogenic index was utilized as a surrogate index of early-phase insulin release [17,18], which was calculated by dividing the increment in serum insulin at 0.5-h by the increment in plasma glucose at 0.5-h of the OGTT (InsAUC_30_/GluAUC_30_). In addition, oral disposition index (oral DI), which assesses whether insulin secretion is augmented sufficiently to compensate for the prevailing degree of insulin resistance, was calculated as the product of 1/fasting insulin × insulinogenic index [17,18]. Reductions in oral DI are predictive of increased risk of progression to diabetes in adults [18].

### Determination of body composition

Body mass index (BMI) was calculated using the Quetelet index (kg/m^2^) [19]. iDXA (GE-Healthcare Madison, WI, software version 15) scan was acquired with a total body scanner in each participant to determine the body composition. iDXA allows the simultaneous measurement of bone mass, fat mass and lean body mass from the ratio of attenuation of two energy beams passing through the body [20]. CoreScan software was used to estimate the visceral fat mass based on the measurement of abdominal and subcutaneous adipose tissues.

### Blood sample collection and measurement

Ten milliliters of blood were drawn from each participant by venipuncture in a tube containing EDTA. The blood was then centrifuged at 3000g for 10min, immediately divided into aliquots, and frozen at -80°C until analysis. Plasma concentrations of leptin (minimum sensitivity=1.04 ng/ml, intra-assay CV=1.13%) and adiponectin (minimum sensitivity=1.24 μg/ml, intra-assay CV=3.98%) were analyzed using commercially available radioimmunoassay kits (Millipore, Billerica, MA). All samples were run in duplicate and the mean of the duplicate samples was reported.

### Postmortem human brain tissue preparation and Western blot analysis

All the human brain tissues used in this study were obtained from the Alabama Brain Bank with the permission from the UAB Brain Collection Steering Committee. All experimental procedures were approved by the UAB Institutional Review Board. For all the postmortem human cases used in this study, two independent psychiatrists established MDD diagnosis using the Structured Clinical Interview for DSM-IV [14] based on the review of all available medical records. Control cases had enough information from next of kin and medical records to exclude any major neuropsychiatric disorders. Consent was obtained from next of kin for each subject. None of cases was diagnosed with diabetes. Two types of brain samples were used in this study, including hypothalamus and hippocampus. Brain tissue was prepared for Western blots as previously described [21]. Briefly, tissue was reconstituted in 5 mM Tris-HCl pH 7.4, 0.32 M sucrose, and a protease inhibitor tablet (Complete Mini, Roche Diagnostics, Mannheim, Germany). Tissue was homogenized using a Power Gen 125 homogenizer (Thermo Fisher Scientific, Rockford, IL) at speed 5 for 60s. Homogenates were assayed for protein concentration using a BCA protein assay kit (Termo Scientific, Rockford, IL), and stored at -80°C until used.

Commercially available antibodies were used for the Western blot analyses, including anti-phospho serine (PS) 473-AKT and total-AKT (Cell Signaling, Danvers, MA). Samples for Western blots were placed in reducing buffer-containing *β*-mercaptoethanol and heated at 70°C for 10min. Samples for each subject were then run in duplicate by SDS-polyacrylamide gel electrophoresis on Invitrogen 4-12% gradient gels (Carlsbad, CA), and transferred to polyvinylidene fluoride membrane using Bio-Rad semi-dry transblotter (Hercules, CA). The membranes were blocked in LiCor (Lincoln, NE) blocking buffer for 1-h at room temperature, and probed with primary antibody in 0.1% Tween LiCor blocking buffer in 1% BSA. Membranes were then washed 4 times for 5min each with 0.01% Tween phosphate-buffered saline. Membranes were probed with IR-dye labeled secondary antibody in 0.1% Tween, 0.01% SDS LiCor blocking buffer for 1-h at room temperature in the dark. Membranes were washed again with 0.01% Tween phosphate-buffered saline 4 times for 5min each and then briefly rinsed 3 times in distilled water. The blots were stored in distilled water at 4°C until scanned using the LiCor Odyssey laser-based image detection method [17]. The blots were quantified using LiCor Odyssey 3.0 analytical software (Lincoln, NE).

### Statistical Analysis

All statistical analyses were performed using the Statistical Package for the Social Sciences version 22 (SPSS Inc., Chicago, IL). All data are presented as mean ± standard error unless otherwise stated. All variables were tested for normality of distribution by means of Kolmogorov-Smirnoff tests. Nonparametric tests were applied for data that were not from a normal distribution. Independent samples *t*-tests were carried out to detect the difference between groups. *P* values <0.05 were considered significant. Correlation analyses were used to estimate the associations between two variables.

## Results

### Participant characteristics

There were no significant differences in the variables including age, race, sex and BMI between the two groups, MDD patients and controls (Table 1). However, the depression scores using the Mini International Neuropsychiatric Interview were significantly different between these two groups (*p*<0.001).

### Glucose responses to the OGTT

**Figure 1:**
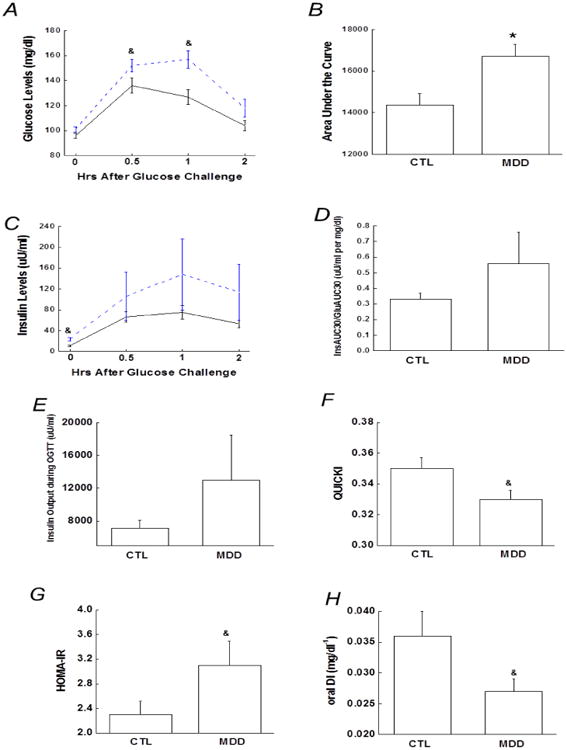
A) 2-h plasma glucose levels during the OGTT. B) Bar graph of glucose area under the curve during the OGTT. C) 2-h insulin levels during the OGTT. D) Early-phase insulin release (InsAUC_30_/GluAUC_30_) during the OGTT. E) Total insulin release or insulin area under the curve during the OGTT. F and G) Surrogate index of insulin sensitivity, including QUICKI and HOMA-IR. H) Oral disposition index (1/fasting insulin × InsAUC_30_/GluAUC_30_) during the OGTT. Data are presented as the mean ± S.E in each group. Dash line stands for depressive group and solid line stands for control group; ^&^, *p*<0.05; compared with control group at the same time point during the OGTT in Figures. A and C. ^&^, *p*<0.05; *, *p*<0.01, compared with controls. CTL, controls; MDD, major depressive disorder.

As shown in Figure 1A, glucose levels rose to higher levels following an oral glucose challenge in the MDD than in controls. While both fasting and 2-h glucose concentrations tended to be higher in the MDD group compared with the controls, the differences were not statistically significant (*p*=0.076, 0.077, respectively). However, 0.5-h and 1-h glucose concentrations were significantly greater in the MDD group than the controls (*p*=0.039 and 0.002, respectively, Figure 1A). Glucose iAUC was also significantly greater in the MDD group (*p*=0.006, Figure 1B), indicative of relative degree of glucose intolerance.

### Insulin responses to the OGTT

During the OGTT, fasting insulin concentrations were higher in the MDD group compared with the controls, whereas insulin levels at 0.5-h, 1-h and 2-h only tended to be higher in the MDD group (*p*=039, 0.29, 0.25, respectively, Figure 1C). Compared with the controls, both the oral glucose-induced early phase insulin response, as determined by the insulinogenic index, and insulin iAUC, denoted as total insulin secretion during the OGTT, tended to be greater in the MDD group, but not significantly (*p*=0.25, 0.15, respectively, Figures 1D and 1E).

### Insulin sensitivity and oral disposition index

Insulin sensitivity, as estimated by the QUICKI and HOMA-IR, was significantly lower in individuals with MDD compared with the controls (Figures 1F and 1G). Correlational analysis revealed that insulin sensitivity as measured by HOMA-IR and QUICKI correlated with the OGTT 1-h glucose (r=0.27, *p*=0.044, and -0.35, *p*=0.004, respectively). The oral DI in the MDD group was lower by ∼25% compared with the controls (Figure 1H).

### Effects of MDD on body composition and adipocyte-derived adipokines

**Figure 2:**
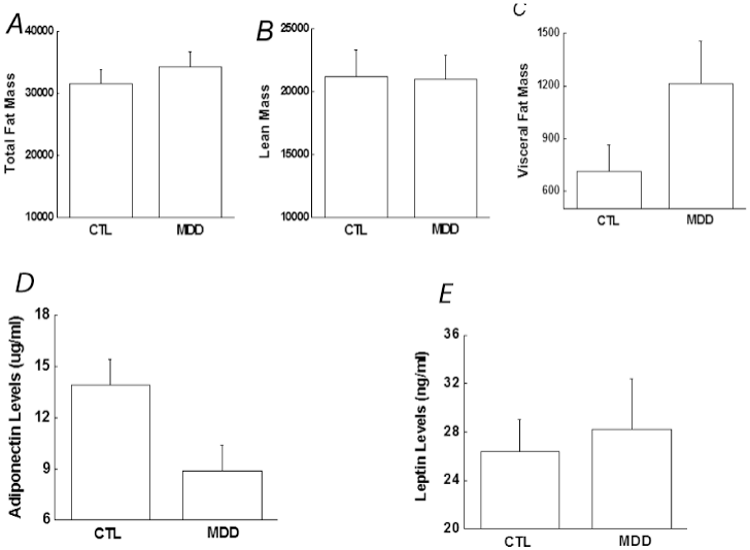
Comparison of body composition and adipokines between the controls and depressive patients, including (A) Total body fat mass, (B) Lean mass, (C) Visceral fat mass, (D) Adiponectin and (E) Leptin. Data are presented as the mean ± S.E in each group. ^&^, *p*<0.05, compared with control group. CTL, controls; MDD, major depressive disorder.

Recent evidence shows that obesity, especially visceral obesity, is associated with insulin resistance and increased risk for developing type 2 diabetes [22]. In our matched groups, BMI did not differ between the MDD and control groups as shown in Table 1, and body lean mass and total fat mass were also similar between the two groups (Figures 2A and 2B). However, visceral fat mass was significantly greater in the MDD group than in the controls (Figure 2C). Regression analysis revealed that visceral fat mass correlated with surrogate indices of insulin sensitivity, including QUICKI (r=-0.34, *p*=0.039).

Pairwise comparison between subjects in the two groups revealed that the MDD group had significantly lower levels of adiponectin (Figure 2D), whereas the two groups exhibited similar levels of leptin (Figure 2E). Furthermore, adiponectin levels correlated with surrogate indices of insulin sensitivity, including QUICKI (r=0.33, *p*=0.032).

### Impaired insulin signaling in hypothalamus and hippocampus in patients with MDD

Postmortem human brain cases were carefully selected with attempts to match as much as possible the cases in the control and MDD groups by their age, race, sex, post-mortem interval (PMI) and brain pH. As shown in Table 2, cases in the two groups did not differ in mean values for age, race, sex, PMI and brain pH.

**Figure 3:**
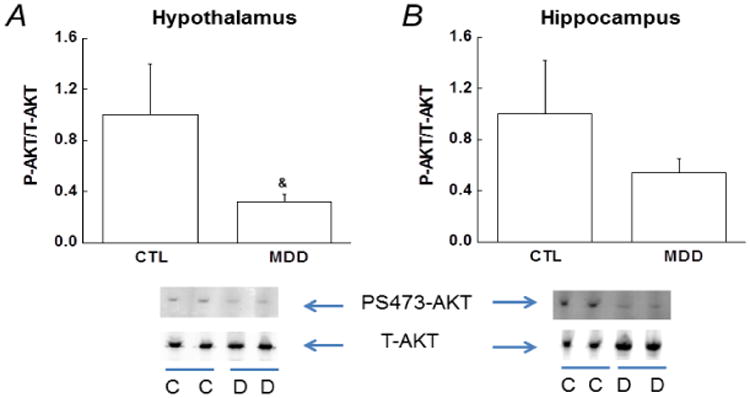
Decreased insulin action via phosphorylated serine-AKT (PS473-AKT) in hypothalamus and hippocampus. Representative Western blots from the controls and depressive patients in hypothalamus (A) and hippocampus (B) are presented below the bar graphs. The ratio between phospho-AKT and total AKT levels in the controls is arbitrarily set to 1, and the PS473-AKT levels in depressive group is normalized by control group and presented in fold change. Data are presented as the mean ± S.E in each group (n=6 per group). ^&^, *p*<0.05, compared with the controls. CTL/C, controls; MDD/D, major depressive disorder.

Changes in insulin signaling pathways were characterized by examining the expression of PS473-AKT (a common and active phosphorylation site in AKT, which is the ultimate protein in the IRS/PI3K/AKT pathway) [13,12] in hypothalamus and hippocampus from the MDD patients and controls. Compared with the controls, Western blot analysis revealed significantly lower PS473-AKT protein levels in the MDD patients in hypothalamus (Figure 3A), but not in hippocampus (Figure 3B), indicating a significant diminution in insulin action in individuals with MDD.

## Discussion

In this cross-sectional study, the main finding was that insulin sensitivity, determined by surrogate measures of HOMA-IR and QUICKI, was considerably lower in the MDD patients. Furthermore, a compensatory increase in insulin release, manifested by increased early-phase insulin release and total insulin secretion during the OGTT although not significantly, was observed to maintain fasting and 2-h glucose levels within the normal range in patients with depression. However, 1-h glucose levels were substantially greater in the MDD patients compared with the controls. In other words, decreased insulin sensitivity, combined with defects in insulin secretion, was responsible for the abnormal glucose tolerance observed in the MDD subjects.

While insulin resistance and the reduction in oral DI place individuals at an increased risk of developing type 2 diabetes, some studies have demonstrated that 1-h glucose during the OGTT could be a better risk predictor for future diabetes than fasting and 2-h glucose in both Mexican-American individuals and European Caucasian populations [8]. In the present study, fasting and 2-h glucose concentrations did not differ significantly between the MDD and controls, however, 1-h glucose concentrations during the OGTT were significantly higher in the MDD. In addition, our study showed that 1-h glucose correlated with OGTT-derived indices of insulin sensitivity. It is known that reduced insulin sensitivity is present long before the onset of diabetes and the characteristic pathophysiologic disturbance is responsible for the development of type 2 diabetes [4,16]. The strong correlation between the 1-h glucose concentration and reduced insulin sensitivity may explain why the 1-h glucose could be a better risk predictor for future type 2 diabetes compared with fasting or 2-h glucose concentrations. Our study emphasizes the important of measuring the 1-h glucose levels and exploring its role in diabetes-related studies.

Adequate insulin action in brain and peripheral tissues is critical to maintain glucose homeostasis. The insulin receptors are widely expressed in the central nervous system including hippocampus and hypothalamus [13]. We examined insulin action by studying the insulin signaling in two postmortem brain regions from the MDD patients and controls, and found that insulin action was impaired in hypothalamus as indicated by reduced PS473-AKT levels. In other words, central insulin action was impaired although none of these 6 depressive patients were diagnosed with diabetes. Our observation of impaired insulin action in postmortem brain tissues is actually supported by recent reports [21-24]. Recent studies have demonstrated that the brain is an insulin-sensitive organ and plays important physiological roles including glucose homeostasis, feeding behavior, body weight and cognitive processes [23,24]. Alterations of central insulin action may contribute to the reduced insulin sensitivity in the MDD patients. However, further investigation is warranted to include more brain areas to examine insulin signaling and to verify the critical role of central insulin action in developing prediabetes and subsequent diabetes in individuals with MDD.

There are multiple mechanisms for decreased insulin sensitivity or insulin resistance, and one of them is related with visceral adiposity [25]. In the present study, visceral fat mass was found to be greatly increased in the MDD patients compared with the controls, and correlated with reduced insulin sensitivity. It has been proposed that the link between visceral adiposity and insulin resistance is probably due to the inflammatory profile in visceral adipose tissue [26,27]. Adipose tissue secretes a number of cytokines, also termed adipokines, including adiponectin and leptin [27,28]. Chronic and low-grade inflammation caused by altered adipokine secretion may alter glucose homeostasis and contribute to the risk for increased visceral fat mass [26,28]. It has been demonstrated that adiponectin exhibits beneficial effects on glucose homeostasis and low level of adiponectin predicts an increased risk for type 2 diabetes through reducing insulin sensitivity [27]. Our study found that adiponectin level was decreased in the MDD patients, whereas leptin level was not. In addition, data analyses showed that insulin sensitivity was also correlated with adiponectin levels, indicating that reduced insulin sensitivity and inflammatory process are related with each other in the MDD patients.

The results of our study raise an interesting question about whether the MDD patients should be screened with the OGTT to identify those susceptible individuals who are at a higher risk for developing prediabetes and subsequent diabetes. Studies suggest that people with reduced insulin sensitivity or prediabetes can prevent or delay the onset for diabetes, and in some cases even return their insulin sensitivity to normal, by lifestyle modifications such as weight loss and physical activity [29]. Given the high mortality and morbidity of diabetes and its complications, prevention seems to be critical for improving health outcomes in individuals with MDD. In other words, an effective strategy could focus on improving insulin sensitivity and/or normalizing 1-h glucose concentrations in the MDD patients through lifestyle modifications.

In addition, we acknowledge several limitations in our study. First, the sample size is relatively small. However, data analyses were completed in 60 participants who were thoroughly investigated. Statistical analyses were kept simple for us to obtain meaningful results in a relative small sample. A second limitation is that we did not investigate the effects of antidepressants on glucose metabolism. Reports from cross-sectional and longitudinal studies have produced inconsistent results in terms of the effects of antidepressants on glucose homeostasis [30]. Inconsistent findings could be due to different antidepressants, study populations, sample sizes and other reasons. Our future studies could focus on the impact of antidepressants on the risk of prediabetes for more evidence. We are also aware that depressive patients in our study are heterogenous in terms of the duration and episodes of depression. A correlational analysis of insulin sensitivity measures with the duration and episodes of depression would be conducted when sample size is expanded, and such a project would obviously complement our current work.

In summary, we have showed that insulin sensitivity is impaired in the MDD patients, together with reductions in the disposition index (i.e., oral DI), resulting in impaired glucose tolerance as manifest by elevated 1-hr glucose values following an oral glucose challenge. Furthermore, the decrease of insulin sensitivity is associated with increased visceral fat mass and reduced adiponectin levels. Finally, levels of phospho-AKT were reduced in brain tissues from MDD patients compared with controls, suggesting that insulin resistance may have affected the central nervous system as well as systemic metabolism.

## Figures and Tables

**Table 1:** Demographic data for participants.

Variables	Controls	MDD
N, Sample size	30	30
Age (years)	39.8 ± 1.9	44.2 ± 2.2
Number of female subjects	21	18
Number of Caucasians	14	16
BMI	28.2 ± 4.3	29.3 ± 5.9
Beck Depression Scale	7 ± 1.2	25.8 ± 2.4

Note: only African Americans and Caucasians were enrolled in this study. MDD: Major Depressive Disorder; BMI: Body Mass Index.

**Table 2:** Demographic data in postmortem cases.

Case	Age (years)	Race	Sex	Post-mortem interval	pH
MDD1	84	C	F	8	6.0
MDD2	49	C	F	10	6.9
MDD3	63	C	F	30	6.3
MDD4	51	C	M	28	5.6
MDD5	50	C	F	12	6.7
MDD6	64	C	M	17	6.2
Mean ± s.e	60.2 ± 5.5			17.5 ± 3.8	6.3 ± 0.2
CTL1	82	C	F	25	5.9
CTL2	32	C	F	8	6.3
CTL3	63	C	F	22	6.1
CTL4	48	C	M	23	5.9
CTL5	58	C	F	24	5.4
CTL6	69	C	M	15	6.1
Mean ± s.e	58.7 ± 7.1			19.5 ± 2.7	5.9 ± 0.1

PMI: Postmortem Interval; MDD: Major Depressive Disorder; CTL: Control; C: Caucasian; F: female; M: male; S.E: standard error.

## References

[1] Abdul-Ghani MA, Williams K, DeFronzo R, Stern M (2006). Risk of progression to type 2 diabetes based on relationship between postload plasma glucose and fasting plasma glucose. Diabetes Care.

[2] Abdul-Ghani MA, Williams K, DeFronzo RA, Stern M (2007). What is the best predictor of future type 2 diabetes?. Diabetes Care.

[3] Barnard K, Peveler RC, Holt RI (2013). Antidepressant medication as a risk factor for type 2 diabetes and impaired glucose regulation: systematic review. Diabetes Care.

[4] Carnethon MR, Kinder LS, Fair JM, Stafford RS, Fortmann SP (2003). Symptoms of depression as a risk factor for incident diabetes: findings from the National Health and Nutrition Examination Epidemiologic Follow-up Study, 1971-1992. Am J Epidemiol.

[5] Cusi K, Maezono K, Osman A, Pendergrass M, Patti ME (2000). Insulin resistance differentially affects the PI 3-kinase- and MAP kinase-mediated signaling in human muscle. J Clin Invest.

[6] Derakhshan F, Toth C (2013). Insulin and the brain. Curr Diabetes Rev.

[7] Ferrannini E, Gastaldelli A, Miyazak Y, Matsuda M, Mari A (2005). beta-Cell function in subjects spanning the range from normal glucose tolerance to overt diabetes: a new analysis. J Clin Endocrinol Metab.

[8] Ferrante AW (2007). Obesity-induced inflammation: a metabolic dialogue in the language of inflammation. J Intern Med.

[9] First MB, Spitzer RL, Gibbon M, Williams JBW (2002). Structured Clinical Interview for DSM-IV-TR Axis I Disorders, Research Version, Patient Edition (SCID-I/P).

[10] Funk AJ, Rumbaugh G, Harotunian V, McCullumsmith RE, Meador-Woodruff JH (2009). Decreased expression of NMDA receptor-associated proteins in frontal cortex of elderly patients with schizophrenia. Neuroreport.

[11] Gabir MM, Hanson RL, Dabelea D, Imperatore G, Roumain J (2000). Plasma glucose and prediction of microvascular disease and mortality: evaluation of 1997 American Diabetes Association and 1999 World Health Organization criteria for diagnosis of diabetes. Diabetes Care.

[12] Garrow JS, Webster J (1985). Quetelet's index (W/H2) as a measure of fatness. Int J Obes.

[13] Gerozissis K (2008). Brain insulin, energy and glucose homeostasis; genes, environment and metabolic pathologies. Eur J Pharmacol.

[14] Gower BA, Hunter GR, Chandler-Laney PC, Alvarez JA, Bush NC (2010). Glucose metabolism and diet predict changes in adiposity and fat distribution in weight-reduced women. Obesity (Silver Spring).

[15] Kessler RC, Berglund P, Demler O, Jin R, Merikangas KR (2005). Lifetime prevalence and age-of-onset distributions of DSM-IV disorders in the National Comorbidity Survey Replication. Arch Gen Psychiatry.

[16] Knol MJ, Twisk JW, Beekman AT, Heine RJ, Snoek FJ (2006). Depression as a risk factor for the onset of type 2 diabetes mellitus. A meta-analysis. Diabetologia.

[17] Lara-Castro C, Fu Y, Chung BH, Garvey WT (2007). Adiponectin and the metabolic syndrome: mechanisms mediating risk for metabolic and cardiovascular disease. Curr Opin Lipidol.

[18] Li L, Li X, Zhou W, Messina JL (2013). Acute psychological stress results in the rapid development of insulin resistance. J Endocrinol.

[19] O'Shaughnessy IM, Myers TJ, Stepniakowski K, Nazzaro P, Kelly TM (1995). Glucose metabolism in abdominally obese hypertensive and normotensive subjects. Hypertension.

[20] Phillips DI, Clark PM, Hales CN, Osmond C (1994). Understanding oral glucose tolerance: comparison of glucose or insulin measurements during the oral glucose tolerance test with specific measurements of insulin resistance and insulin secretion. Diabet Med.

[21] Paneni F, Costantino S, Volpe M, Lüscher TF, Cosentino F (2013). Epigenetic signatures and vascular risk in type 2 diabetes: a clinical perspective. Atherosclerosis.

[22] Ross R, Aru J, Freeman J, Hudson R, Janssen I (2002). Abdominal adiposity and insulin resistance in obese men. Am J Physiol Endocrinol Metab.

[23] Sheehan DV, Lecrubier Y, Sheehan KH, Amorim P, Janavs J (1998). The Mini-International Neuropsychiatric Interview (M.I.N.I.): the development and validation of a structured diagnostic psychiatric interview for DSM-IV and ICD-10. J Clin Psychiatry.

[24] Sinha MK, Caro JF (1998). Clinical aspects of leptin. Vitam Horm.

[25] Tuomilehto J, Lindstratm J, Eriksson JG, Valle TT, Haomaolaoinen H (2001). Finnish Diabetes Prevention Study Group. Prevention of type 2 diabetes mellitus by changes in lifestyle among subjects with impaired glucose tolerance. N Engl J Med.

[26] Utzschneider KM, Prigeon RL, Faulenbach MV, Tong J, Carr DB (2009). Oral disposition index predicts the development of future diabetes above and beyond fasting and 2-h glucose levels. Diabetes Care.

[27] Weber B, Schweiger U, Deuschle M, Heuser I (2000). Major depression and impaired glucose tolerance. Exp Clin Endocrinol Diabetes.

[28] Weyer C, Bogardus C, Mott DM, Pratley RE (1999). The natural history of insulin secretory dysfunction and insulin resistance in the pathogenesis of type 2 diabetes mellitus. J Clin Invest.

[29] Weyer C, Bogardus C, Pratley RE (1999). Metabolic characteristics of individuals with impaired fasting glucose and/or impaired glucose tolerance. Diabetes.

[30] Winokur A, Maislin G, Phillips JL, Amsterdam JD (1988). Insulin resistance after oral glucose tolerance testing in patients with major depression. Am J Psychiatry.

